# Preparation Strategies of the Anti-Mycobacterial Drug Bedaquiline for Intrapulmonary Routes of Administration

**DOI:** 10.3390/ph16050729

**Published:** 2023-05-11

**Authors:** Sara E. Maloney, Ian E. Stewart, Brendan K. Podell, Hadley E. Gary, Jeffrey B. Mecham, Bryan J. Berube, Susan L. Baldwin, Rhea N. Coler, Anthony J. Hickey

**Affiliations:** 1 Technology Advancement and Commercialization, RTI International, Research Triangle Park, NC 27709, USA; 2 Mycobacteria Research Laboratories, Department of Microbiology, Immunology, and Pathology, Colorado State University, Fort Collins, CO 80523, USA; 3 Seattle Children’s Research Institute, Center for Global Infectious Disease Research, Seattle, WA 98109, USA; 4 Department of Pediatrics, University of Washington School of Medicine, Seattle, WA 98195, USA; 5 Department of Global Health, University of Washington, Seattle, WA 98195, USA

**Keywords:** bedaquiline, tuberculosis, inhalation therapy, spray drying, dry powder inhaler, excipient

## Abstract

*Mycobacterium tuberculosis* (*M.tb*) has infected one-quarter of the world’s population and led to the deaths of 1.6 million individuals in 2021 according to estimates from the World Health Organization. The rise in prevalence of multidrug-resistant and extensively drug-resistant *M.tb* strains coupled with insufficient therapies to treat such strains has motivated the development of more effective treatments and/or delivery modalities. Bedaquiline, a diarylquinoline antimycobacterial agent, effectively targets mycobacterial ATP synthase but may lead to systemic complications upon oral delivery. Targeted delivery of bedaquiline to the lungs represents an alternative strategy to harness the sterilizing benefits of the drug against *M.tb* while mitigating off-target side effects. Two pulmonary delivery modalities were developed herein, including dry powder inhalation and liquid instillation. Despite bedaquiline’s poor water solubility, spray drying was performed in predominantly aqueous conditions (≥80%) to avoid a closed-loop, inert system. Aerosols of spray-dried bedaquiline with L-leucine excipient outperformed spray-dried bedaquiline alone, demonstrating superior fine particle fraction metrics (~89% of the emitted dose below <5 µm), suitable for inhalation therapies. Furthermore, the use of a 2-hydroxypropyl-β-cyclodextrin excipient allowed a molecular dispersion of bedaquiline in an aqueous solution for liquid instillation. Both delivery modalities were successfully administered to Hartley guinea pigs for pharmacokinetic analysis and were well-tolerated by the animals. Intrapulmonary liquid delivery of bedaquiline led to adequate serum absorption and appropriate peak serum concentrations of the drug. The liquid formulation was superior in systemic uptake compared to the powder formulation. The predominant route via which *M.tb* bacilli enter the body is aerosol droplets that are deposited onto airway surfaces. For this reason, we believe that further studies should focus on inhalation or intrapulmonary therapies that target the site of entry and primary site of infection for *M.tb*.

## 1. Introduction

In 2021, *Mycobacterium tuberculosis* (*M.tb*) infected approximately 10.6 million people, and 1.6 million deaths were attributed to infection with *M.tb* [1]. Indeed, tuberculosis (TB) is the second leading cause of death due to infectious disease, only recently surpassed by COVID-19 [1]. Complicating treatment regimens for patients with TB, approximately 3.7% of new TB patients have multidrug-resistant TB (MDR-TB). Furthermore, strains of MDR-TB are on the rise worldwide [1]. MDR-TB is caused by bacteria that do not respond to isoniazid and rifampicin, the two most effective first-line anti-tuberculosis drugs [1,2,3,4]. Of the patients with MDR-TB, 9% have extensively drug-resistant TB (XDR-TB), which is resistant to isoniazid, rifampicin, any fluoroquinolone, and any second-line injectable agent (i.e., capreomycin, kanamycin, or amikacin) [2,3,4]. The treatment success rate for MDR-TB was only 60% in 2019, according to the World Health Organization [1]. More effective treatments are therefore required to prevent the transmission of *M.tb*.

Bedaquiline (TMC207 [BDQ]), a novel oral diarylquinoline antimycobacterial agent, was approved by the Food and Drug Administration (FDA) in 2012, making it one of the newest drugs used for the treatment of MDR/XDR-TB. BDQ exerts bactericidal and sterilizing activity against *M.tb* by inhibiting the proton pump of mycobacterial ATP synthase [2,5,6,7]. Importantly, BDQ is effective at inhibiting both actively replicating and dormant cells, such as those present in latent TB infection [6]. BDQ has a narrow spectrum of activity against mycobacteria, with its efficacy unaltered by the presence of resistance mechanisms to other anti-TB drugs [2,6,7]. Multiple clinical trials have highlighted the benefit of incorporating BDQ into the standard second-line anti-TB regiment in MDR/XDR-TB-positive patients. In such trials, the addition of BDQ to standard therapy reduced the time to conversion to a TB-negative sputum culture [5,8,9]. The most frequent adverse effects of oral BDQ delivery were nausea, vomiting, and arthralgia. However, other serious side effects have been associated with oral delivery of BDQ, including increased liver aminotransferase levels and QTc prolongation [6,8,10]. Of utmost concern, more deaths have been reported in the oral BDQ group as compared to the placebo group in one study, with a mortality rate of 11.4% and 2.5% for the BDQ and placebo groups, respectively [8,10]. This disparity has not been attributed to any cause. As a result of these concerns, BDQ is currently only approved to treat adults with pulmonary MDR-TB in combination with other anti-TB drugs when an effective treatment regimen cannot be provided otherwise [2].

Irrespective of BDQ’s demonstrated efficacy, the limitations imposed by adverse reactions and unexplained increased mortality rates hinder its potential in treating MDR/XDR-TB patients [7]. Targeted delivery of BDQ locally to the lungs via inhalation rather than systemically via oral delivery may help alleviate some of these side effects by reducing the required dose, which in turn, may increase patient compliance [11,12,13]. Dry powder inhalers (DPIs) represent an attractive delivery modality, as administering BDQ via DPI may increase the availability of the drug to more rural areas as the solid-state drug product can often be stored at room temperature and will not need to be administered via healthcare professionals (i.e., injectables).

Previously, chitosan and BDQ nanoparticles (~110 nm) were prepared by freeze-drying and lactose blending [14]. However, the use of lactose carrier particles limits the achievable dose of BDQ. As an alternative approach, spray drying, a well-established method for preparing respirable particles, has been employed [15]. Spray-dried BDQ alone exhibited a low yield and insufficient fine particle fraction with respect to the emitted dose (FPF_ED_, 31.3%) [4,16]. Combination with leucine as an excipient (20 wt%) increased the FPF_ED_ to 74.4% [16]. Further combination of BDQ with leucine and pyrazinamide or leucine and moxifloxacin and pyrazinamide resulted in FPF_ED_ metrics of >66% and >75%, respectively [4,17]. However, in all cases, a closed loop, inert system for the spray dryer was required for the manufacture of BDQ microparticles due to its water insolubility.

Herein, we modified existing spray drying procedures for BDQ manufacture with and without leucine excipient to eliminate the need for a closed-loop, inert system. The role of leucine as an excipient was examined following spray drying, demonstrating strong benefits in aerodynamic performance upon its inclusion. The optimal spray-dried powder was also used to enhance BDQ water solubility to expand dosing in the form of a fully aqueous solution. Lastly, the spray-dried BDQ powder containing leucine was administered to healthy guinea pigs as an aerosol using custom dosators and by liquid instillation of an aqueous BDQ solution for pharmacokinetic (PK) analysis, with liquid instillation yielding greater serum concentrations of BDQ than powder delivery.

## 2. Results and Discussion

### 2.1. Powder Manufacture and Particle Morphology

Bedaquiline fumarate is insoluble in an aqueous solution [18], presenting challenges for spray drying without the incorporation of an inert loop. Without such additional equipment, the use of organic solvent is limited to 20 vol% for the precursor solution using a Buchi B-290 spray dryer [19]. Solvents including 20 vol% ethanol, methanol, isopropanol, or dimethylsulfoxide (DMSO) were first evaluated in combination with 80 vol% deionized water. However, the addition of BDQ to 80:20 water:ethanol, water:isopropanol, or water:methanol did not result in dissolution. The addition of glacial acetic acid (10 vol%) to 70 vol% water and 20 vol% ethanol did facilitate the solubility of BDQ. However, such acidic conditions are not ideal for spray drying equipment without specialized acid-resistant materials [19]. Reducing glacial acetic acid concentrations to 0.01 vol% for a pH more conducive to spray drying resulted in poor solubility of BDQ and clogging of the spray drying nozzle. BDQ was soluble in 80:10:10 water:DMSO:ethanol when the pH was adjusted to 2.5–3 with acetic acid. Unfortunately, it was evident that the DMSO content of the solvent system was not fully evaporating, leaving droplets of liquid in the collection vessel. A greater temperature was likely needed for DMSO to be utilized; however, the degradation of BDQ begins at ~190 °C, limiting the inlet temperature of the spray dryer to ~170 °C to avoid active pharmaceutical ingredient (API) degradation during spray drying. Ultimately, BDQ was soluble at 2.35 mg/mL in a solvent system of 80:10:10 water:ethanol: *N*,*N*-dimethylformamide (DMF) without requiring a decreased pH. Powder recovery ratios for spray drying BDQ (with or without leucine) in this solvent system were 35–40% relative to the total starting mass dissolved in the solution for spray drying. This recovery is in line with previous reports that performed spray drying of BDQ in organic solvent using an inert loop [4,16].

**Figure 1:**
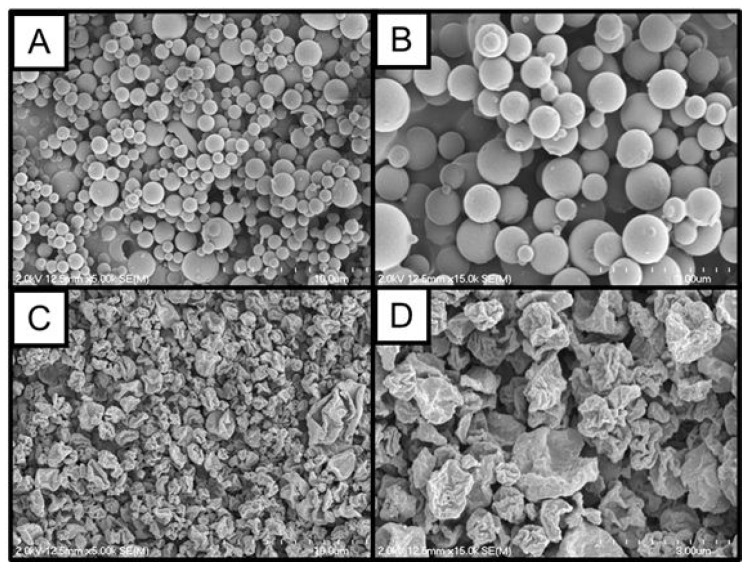
Representative SEM images of (**A**,**B**) SQ-BDQ and (**C**,**D**) SD-BDQ-LEU powders. Magnifications shown are 5000× (**A**,**C**) and 15,000× (**B**,**D**).

BDQ spray dried alone (SD-BDQ) exhibited spherical morphology, visualized in Figure 1A,B, and a geometric diameter of 0.88 ± 0.44 µm. Bedaquiline fumarate was then co-spray dried with L-leucine, an amino acid excipient known to significantly enhance the aerosolization and physical stability of spray-dried powders due to its slightly hydrophobic, surfactant-like nature [20,21,22,23]. Previous reports have noted that the concentration of leucine required for sufficient surface coverage to improve aerosolization is between 10 and 20 wt% [20]. As a result, 15 wt% leucine (balance BDQ) was incorporated into the precursor bedaquiline fumarate solution for spray drying, resulting in SD-BDQ-LEU particles. SD-BDQ-LEU particles had a geometric diameter of 1.00 ± 0.44 µm. The SD-BDQ-LEU particles exhibited collapsed, hollow particle morphology as has been found to minimize contact points between particles, reducing electrostatic cohesion and enhancing deaggregation and dispersibility (Figure 1C,D) [24,25,26].

### 2.2. Physicochemical Characterization of Spray-Dried BDQ Powder

**Figure 2:**
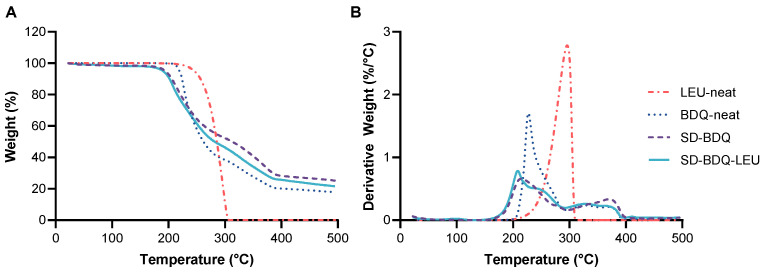
(**A**) Representative thermogram curves of weight with respect to temperature and (**B**) derivative of weight loss vs. temperature curves for leucine (dot-dash, coral), bedaquiline fumarate API as received (dot, blue), SD-BDQ (purple, dash), and SD-BDQ-LEU (solid, teal).

Leucine, BDQ, SD-BDQ, and SD-BDQ-LEU were evaluated using thermogravimetric analysis (TGA) to monitor moisture content and degradation properties (Figure 2A). The weight percent of unbound and bound moisture is determined based on weight loss up to ~100 °C and ~150 °C, respectively. Leucine and BDQ, as received, both exhibited no weight loss up to 150 °C, indicating negligible moisture content. A slight increase in moisture content for the spray-dried particles as compared to the raw API and excipient was expected, as spray-dried particles retain some water from the spray-drying process and are also susceptible to water uptake upon storage [15,27,28]. Upon spray drying, SD-BDQ and SD-BDQ-LEU contained 1.6% and 1.9% moisture, respectively. However, this moisture content was lower than many spray-dried formulations reported in the literature [29,30,31,32], likely due to BDQ’s hydrophobic nature. Furthermore, aggregation due to water content was also less likely to be a concern with BDQ than with a hydrophilic drug.

Leucine alone had an onset of degradation at 184 °C that culminated at 310 °C. The derivative plot of leucine displays a single peak at 296 °C (Figure 2B). BDQ, as received, begins degrading at ~190 °C and exhibits three major degradation events as determined by peaks in the derivative curve at 227 °C (with a minor shoulder at 258 °C), 328 °C, and 368 °C. Upon spray drying, the SD-BDQ and SD-BDQ-LEU powders begin degrading at a lower temperature of 135–140 °C. SD-BDQ displays major degradation events at 215 °C (with a minor shoulder at 274 °C), 326 °C, and 371 °C. SD-BDQ-LEU exhibits major degradation events at 208 °C (with a prominent shoulder at 240 °C), 326 °C, and 363 °C. With the exception of the shift of the initial degradation peak to a lower temperature for both SD-BDQ and SD-BDQ-LEU, the degradation profile at higher temperatures (>300 °C) is similar for BDQ API, SD-BDQ, and SD-BDQ-LEU (Figure 2B). The more rapid degradation onset is likely due to changes in the crystalline structure upon spray drying.

**Figure 3:**
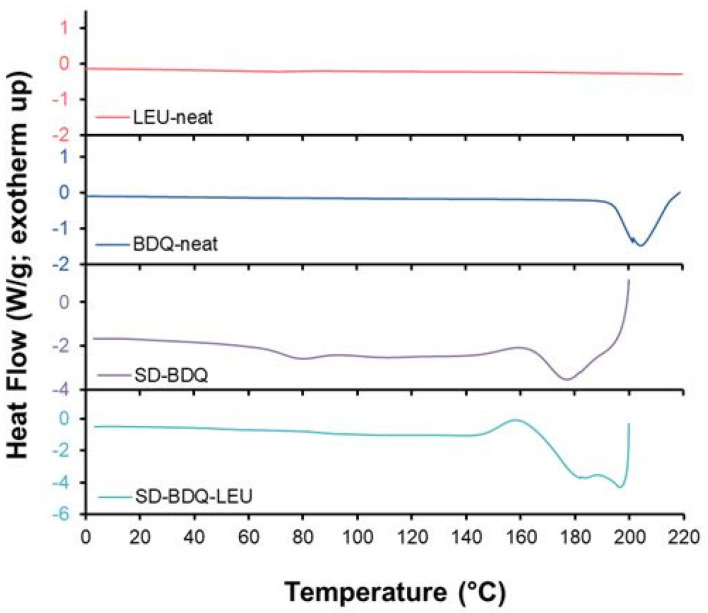
Representative DSC thermograms, from top to bottom, for leucine as received (coral), bedaquiline fumarate API as received (blue), SD-BDQ (purple), and SD-BDQ-LEU (teal). Of note, SD-BDQ and SD-BDQ-LEU analysis was terminated at 200 °C due to lower decomposition temperature.

Crystallinity was first examined using DSC, which identifies molecular structure transition temperatures, with resulting thermograms presented in Figure 3. Heating events were negligible for leucine, which was flat over the full heating profile. BDQ, as received, exhibited an endothermic event from 185 to 215 °C, likely attributed to melting prior to degradation/oxidation beginning at 215 °C. This is in line with previous literature that reports the melting point of bedaquiline fumarate as ~185 °C [18,33]. For both spray-dried powders, this melting event occurs at a lower temperature than for BDQ alone, which is then followed by a degradation/oxidation onset at a lower temperature, as was observed with TGA. Specifically, SD-BDQ exhibited an exothermic peak at 160 °C followed by an endothermic event from 165 to 190 °C. Similarly, SD-BDQ-LEU exhibited an exothermic peak at 158 °C followed by endothermic events spanning 163 to 200 °C. As an important consideration for storage conditions, the glass transition temperature (Tg) was determined for each of the two spray-dried powders [34,35]. The Tg for SD-BDQ and SD-BDQ-LEU was 73 °C and 87 °C, respectively, with the incorporation of the excipient leucine desirably increasing the Tg. Previous literature indicates that amorphous powders for DPIs retain physical stability when storage is at a temperature of at least 40–50 °C below the Tg [36]. Based on the Tg of SD-BDQ and SD-BDQ-LEU, storage at room temperature, as is ideal due to the cost and inconvenience associated with cold-chain storage, is likely to support physical stability [36].

**Figure 4:**
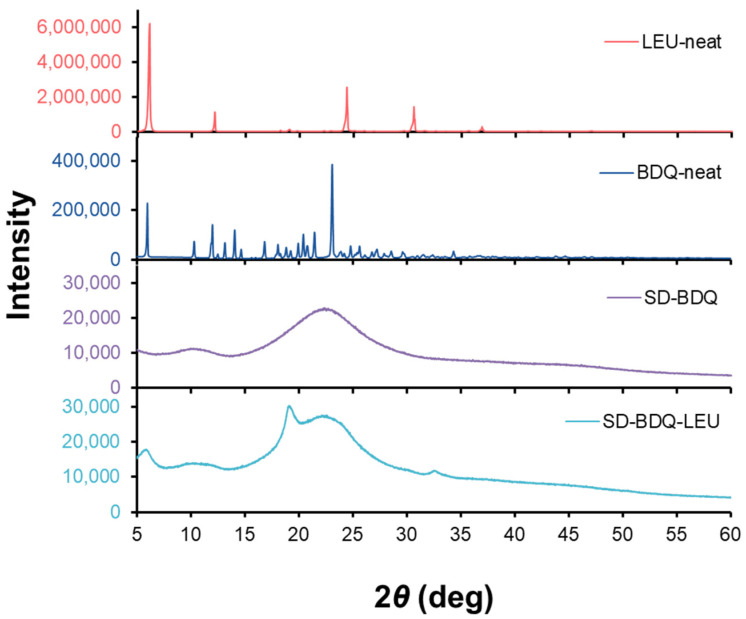
XRPD patterns, from top to bottom, for leucine as received (coral), bedaquiline fumarate API as received (blue), SD-BDQ (purple), and SD-BDQ-LEU (teal).

Crystallinity was then further explored directly using X-ray powder diffraction (XRPD) (Figure 4), a tool to identify atomic structure within the array of molecules in ordered (crystalline) or disordered (amorphous) powders. XRPD patterns revealed crystalline structures for both leucine and BDQ API as received. Upon spray drying, SD-BDQ powders were fully amorphous. SD-BDQ-LEU powders were predominantly amorphous, with small peaks found at 6°, 29.5°, and 33°, attributed to crystalline leucine content on the surface of the particles. The conversion to a mainly amorphous powder from initially crystalline components supports the earlier onset temperature for thermal degradation observed with TGA. Amorphous powders are desired as dry powder inhaler aerosols, as their decreased structural order supports faster dissolution and enhanced bioavailability as compared to crystalline materials [27].

### 2.3. Aerodynamic Properties of Spray-Dried BDQ Powder

**Figure 5:**
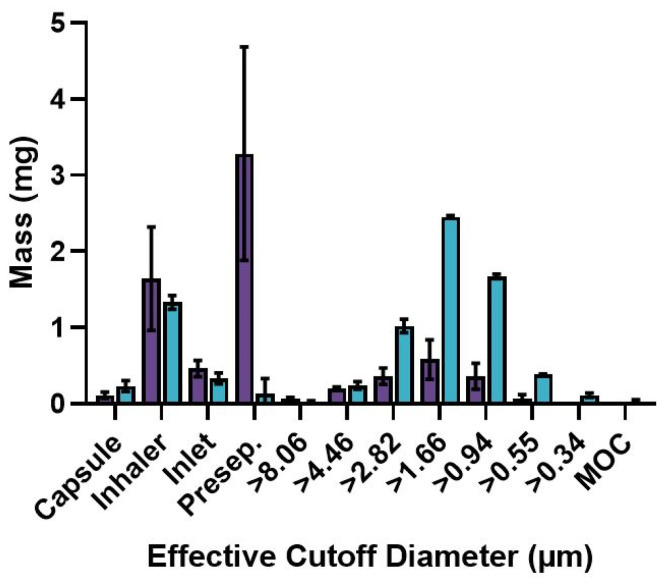
Mass distribution following actuation of 10 mg SD-BDQ (purple) or SD-BDQ-LEU (teal) via RS01 inhaler. The data presented represents the mean ± standard deviation of *n* = 3 analyses. The NGI was operated at an airflow of 60 L/min for 4 s.

SD-BDQ and SD-BDQ-LEU were analyzed using a Next Generation Impactor (NGI) following actuation of an RS01 inhaler at 60 L/min for 4 s. Figure 5 displays the resulting mass distribution of the two powders. SD-BDQ and SD-BDQ-LEU exhibited MMADs of 2.86 ± 0.69 µm and 1.76 ± 0.16 µm, respectively, with both having a relatively narrow size distribution (GSD = 1.79 ± 0.07 and 1.75 ± 0.05 for SD-BDQ and SD-BDQ-LEU, respectively). The use of a predominantly aqueous solution rather than a predominantly organic spray drying solution facilitated the preparation of smaller particles than those reported previously [4,16]. Using a 90:10 EtOH:water spray drying solvent system and an inert loop, Momin et al. reported SD-BDQ particles with an MMAD of 5.9 ± 0.1 µm (GSD = 2.7 ± 0.1) and SD-BDQ-LEU particles (20 wt% leucine) of 2.4 ± 0.2 µm (GSD = 2.0 ± 0.1) [4,16]. Here, the FPFs for SD-BDQ and SD-BDQ-LEU were FPF_N_ = 13.0 ± 6.7% and 55.5 ± 1.8% and FPF_ED_ = 25.2 ± 14.0% and 88.8 ± 4.4%, respectively. The addition of leucine as an excipient at 15 wt% highly influences the resulting distribution of the powders (Figure 5). Only 0.13 ± 0.20 mg of SD-BDQ-LEU was collected in the pre-separator compared to 3.28 ± 1.40 mg of SD-BDQ, highlighting the aggregating nature of the SD-BDQ particles. This discrepancy between BDQ formulations with and without leucine corresponds to previous literature using a predominantly organic system with an inert loop. BDQ-only spray-dried particles were previously reported to have an FPF_ED_ of 31.3%, whereas that co-spray dried with leucine exhibited an FPF_ED_ of 74.4% [16]. The data presented here highlight that almost 90% of the emitted dose of the SD-BDQ-LEU particles is potentially respirable (<5 µm) and is greater than FPF_ED_ metrics reported previously for spray-dried BDQ formulations that used a predominantly organic solvent preparation process [4,16,17]. Combination systems of BDQ/pyrazinamide and BDQ/moxifloxacin/pyrazinamide, both prepared using an inert loop, exhibited FPF_ED_ values of >66% and >75%, respectively [4,17]. Transitioning to a predominantly aqueous solvent system resulted in smaller MMADs and increased FPFs for SD-BDQ-LEU without the need for specialized equipment (i.e., no need for an inert loop).

### 2.4. Preparation of Aqueous BDQ Solution

To expand dosing options, an aqueous solution that could be delivered via the intrapulmonary route for localized delivery to the lung was also investigated. Unsurprisingly, the water insolubility of BDQ made dissolution in an aqueous matrix challenging. To account for these challenges, first, the use of SD-BDQ-LEU rather than BDQ API allowed for increased dissolution owing to the predominantly amorphous structure of the powder. Second, the incorporation of 36% *w*/*v* 2-hydroxypropyl-β-cyclodextrin as an excipient allowed for enhanced solubility of the hydrophobic drug, as has been reported previously [37], allowing the concentration of BDQ in solution to reach 15 mg/mL. 2-Hydroxypropyl-β-cyclodextrin is often used for solution preparation of hydrophobic drugs, as its hydrophobic cavity allows for encapsulation of the drug, while its hydrophilic exterior promotes dissolution in aqueous solution [38,39]. Lastly, sucrose (29% *w*/*v*) was included to increase palatability to the animals [40].

### 2.5. Stability of BDQ Formulations

The stability of the two formulations was evaluated using high-performance liquid chromatography (HPLC) over 7 days. SD-BDQ-LEU powder was stored at room temperature with desiccant. The assay content of bedaquiline in the powder was 66.0%, 65.3%, and 65.0% on days 0, 2, and 7, respectively. These assay contents are near the theoretical bedaquiline content of 70%, accounting for leucine and fumarate content, and show no distinct change over the 7-d span. Bedaquiline aqueous solution was prepared to theoretically contain 15 mg/mL and was stored at 4 °C. The assay content of bedaquiline in the solution was 15.3, 15.0, and 14.9 mg/mL on days 0, 2, and 7, respectively. As with the spray-dried powder, no distinct changes were observed over the 7-d span. Importantly, chromatographic purity was maintained at >96% for all measurements, indicating minimal degradation products. This study demonstrates that both formulations of BDQ were stable over 7 d when stored at either room temperature (~20 °C) or refrigerated (4 °C) for powder or liquid formulations, respectively.

### 2.6. In Vivo Pharmacokinetic Comparison of Liquid and Powder Delivery of BDQ

**Figure 6:**
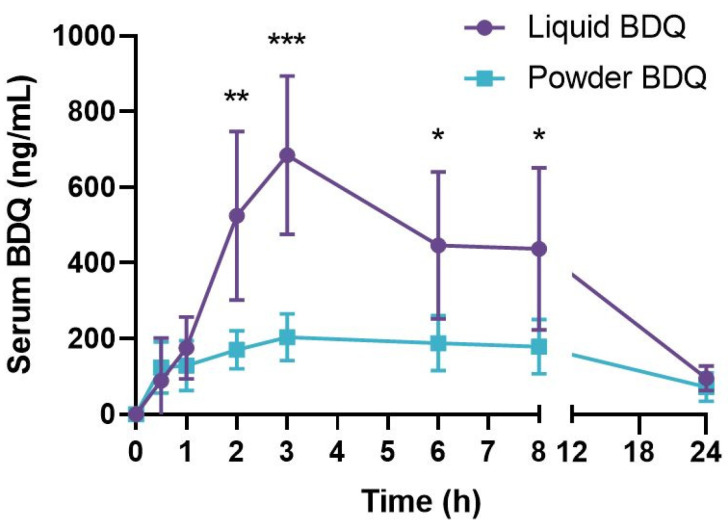
Serum concentration of bedaquiline after intrapulmonary delivery with a single dose of BDQ solution (purple circles) or SD-BDQ-LEU spray-dried powder (teal squares) to cannulated guinea pigs. Both liquid BDQ and powder BDQ were dosed at approximately 15 mg/kg. Error bars represent the standard deviation of *n* = 5 guinea pigs. * *p* < 0.05, ** *p* < 0.01, *** *p* < 0.005.

BDQ, either as a spray-dried powder alone or reconstituted in an aqueous solution, was delivered to guinea pigs through the tracheal lumen at a dose of approximately 15 mg/kg nominal BDQ using either a custom powder dosator [41] or Penn Century microsprayer device, respectively. Serum was collected at intervals following drug delivery for analysis of BDQ content via LC/MS (Figure 6). The area under the curve for the liquid and the spray-dried powder was 7878 ng×h/mL and 3397 ng×h/mL, respectively. The maximum concentrations of the liquid and spray-dried powder in the serum were 684 ± 209 ng/mL and 204 ± 62 ng/mL, respectively, at 3 h post-delivery. At 2 h, 3 h, 6 h, and 8 h after intrapulmonary delivery, significantly more BDQ was recovered in the serum of guinea pigs treated with the drug in solution compared to those treated with the dry powder. The observed pharmacokinetic differences between liquid and powder formulations might be explained by variations inherent in either the formulation or the delivery method. There is the potential for variability in the deposition of the powder formulation in the lungs of anesthetized guinea pigs. It is possible that improved peak serum concentrations would be observed with uniform delivery of the powder formulation. In addition, following dry powder delivery, biological variability may occur due to clearance by mucociliary transport or alveolar macrophage uptake. Consequently, lung exposure to drugs may not be accurately reflected by serum concentrations. As such, further studies will be necessary to ascertain the localized dose of BDQ following liquid- or powder-based delivery.

## 3. Materials and Methods

### 3.1. Materials

Bedaquiline fumarate (BDQ) was purchased from BOC Sciences (Shirley, NY, USA). 2-hydroxypropyl-β-cyclodextrin was purchased from Cayman Chemical Company (Ann Arbor, MI, USA). L-leucine (99%) was purchased from Alfa Aesar (Ward Hill, MA, USA). Sucrose, common lab salts, and common solvents were purchased from Thermo Fisher Scientific (Waltham, MA, USA). Heparin for maintaining intravenous catheter patency was purchased from APP Pharmaceuticals (Schaumburg, IL, USA). Unless otherwise specified, all reagents were used as received without further purification. Nitrogen (N2) gas cylinders were purchased from Airgas National Welders (Raleigh, NC, USA).

### 3.2. Preparation of BDQ Dry Powder Aerosol

Bedaquiline fumarate (BDQ) was dissolved with or without leucine (100:0 or 85:15 BDQ:LEU) at a total solids concentration of 2.35 mg mL^−1^ in 80:10:10 DI water:EtOH:DMF. The solution was spray dried using a Buchi B-290 spray dryer with a high-efficiency cyclone and two-fluid nozzle (inner orifice = 0.7 mm, outer orifice = 1.5 mm). Spray drying parameters included an inlet temperature, aspirator, liquid feed rate, and N2 spray gas rate of 170 °C, 90%, 3 mL min^−1^, and 1744 L h^−1^, respectively. This resulted in an outlet temperature of 75–80 °C. Additionally, 80:20 DI water:DMF was run through the system for 60 s every 20 min. A dehumidifier was placed adjacent to the B-290 air inlet, where room temperature and relative humidity were maintained at 24–27 °C and 20–25%, respectively. The spray-dried powders were collected and stored at room temperature with desiccant.

### 3.3. Morphological Characterization

Spray-dried powders were deposited on an aluminum stub with adhered double-sided carbon tape. The stubs and powders were coated with 10–12 nm of Au/Pd using a Cressington 108 Auto Sputter Coater (Watford, UK). Scanning electron microscopy (SEM) images were captured using a Hitachi S-4700 cold cathode field emission SEM (Schaumburg, IL, USA). The geometric diameter of the spray-dried powder was determined by measuring 600 individual particles using ImageJ software (National Institutes of Health, Bethesda, MD, USA).

### 3.4. Thermal Analysis

Thermogravimetric analysis (TGA) was performed on a Q50 instrument (TA Instruments, New Castle, DE, USA). Small powder masses (~10 mg) were deposited on a platinum TGA sample holder. The TGA experiments were performed in a nitrogen gas environment with a ramp rate of 10 °C min^−1^ from 25 °C to 500 °C. Moisture content was determined as the mass lost up to 150 °C as a result of bound and unbound moisture. Thermograms were processed using TA Universal Analysis software.

### 3.5. Crystallinity

X-ray powder diffraction (D8, Bruker AXS Inc., Karlsruhe, Germany) was conducted by loading a mass of particles on a flat, low-diffraction, silicon wafer and scanning from 5° to 60° 2*θ* at intervals of 0.02° 2*θ* with a 1 s dwell time at 40 kV and 40 mA using a copper anode beam source (0.154 nm wavelength). Diffraction patterns were processed and analyzed with Jade software version 9.6. Differential scanning calorimetry (DSC) was performed using a TA Instruments Q200 DSC (New Castle, DE, USA). Analysis was performed in the range of 0 °C to 220 °C (with the exception of SD-BDQ and SQ-BDQ-LEU, which were run from 0 °C to 200 °C due to lower temperature of decomposition) at a scan rate of 10 °C min^−1^ with powder (5–7 mg) loaded into crimped, aluminum pans with Hermetic lids.

### 3.6. Aerodynamic Performance Characterization

The aerodynamic particle size distribution (APSD) of the spray-dried powder was determined using a next-generation impactor (NGI) operated at 60 L/min for 4 s for each RS01 actuation. Spray-dried powder (10 mg) was loaded into #3 HPMC capsules, which were then loaded into RS01 inhalers (RS01 mod 7, low resistance; Berry, Evansville, IN, USA) and drawn into the NGI using a solenoid-controlled vacuum. Stages of the NGI were pre-coated with 1% *w*/*v* silicone oil in hexane. Impaction runs were performed in triplicate. The powder from the capsule, inhaler, inlet, seven stages, and MOC were collected into 50:50 EtOH:water. The pre-separator was pre-filled with 10 mL of the collection solvent. All solutions were analyzed via UV-Vis (SynergyMX, Biotech) at 285 nm for quantification. The spray-dried powders were utilized for the respective calibration curves to account for the API content in the final powder.

The cumulative mass of particles deposited on the NGI stages was plotted against their corresponding cutoff diameters, conforming to a log-linear distribution on a probability scale. The median of the plot provides the mass median aerodynamic diameter (MMAD). The geometric standard deviation (GSD) was calculated as the square root of the ratio of particle size at the 84th percentile (1 standard deviation above the median) and 16th percentile (1 standard deviation below the median) of the distribution. Impaction results were then used to calculate the fine particle fraction (FPF), which is the percentage of powder collected from stage 3 of the NGI to the micro-orifice collector (particle size < 4.46 µm). The FPF is reported with respect to either the nominal dose (i.e., the dose loaded into the capsule) as the FPF_N_ or the emitted dose (i.e., the mass that reached the inlet of the NGI and below) as the FPF_ED_.

### 3.7. Dosator Preparation

Custom dosators were adapted from those previously reported [29,41] to allow for two actuations of spray-dried 85:15 BDQ-fumarate:LEU (SD-BDQ-LEU). Briefly, a male luer lock to female luer coupler adapter was attached to a 20 G PTFE needle. Screens were made from stainless steel wire cloth, 42 × 42 mesh with a wire diameter of 0.0055”, which was die cut to an outer diameter of 5 mm and placed in the luer adapter. SD-BDQ-LEU (11–12 mg, corresponding to ~7.5 mg BDQ) was loaded into the adapter/needle from the top side of the adapter. A three-way stopcock with a swivel luer lock was then gently attached to the adapter, with care taken not to compact the powder. Two 3-mL syringes were pre-filled with 3 mL of air and attached to the stopcock. The dosator was actuated twice by rotating the stopcock valve for each syringe.

### 3.8. Preparation of Aqueous BDQ Solution

SD-BDQ-LEU was utilized to prepare the liquid BDQ solution to enhance the solubility of BDQ. The prepared solution contained 105 mg of SD-BDQ-LEU powder in 5 mL of 36% *w*/*v* 2-hydroxypropyl-β-cyclodextrin and 29% *w*/*v* sucrose in water, equating to a BDQ concentration of 15 mg/mL. Briefly, the solution was prepared in an amber scintillation vial by first adding ~4 mL DI water (from 5 mL total water volume) and 1.44 g sucrose. The solution was stirred at 35 °C via magnetic stirring. Once dissolved, 1.80 g 2-hydroxypropyl-β-cyclodextrin was added, and the solution was placed in a bath sonicator set to 37 °C for 15–30 min, or as long as needed to dissolve the 2-hydroxypropyl-β-cyclodextrin. Once dissolved, 1.0 M HCl was added to bring the pH to ~1. SD-BDQ-LEU (105 mg) was then added, with the remaining DI water (1 mL) used to aid in the transfer of the powder, and the solution was sonicated for 10–20 min at 37 °C, or as long as needed to dissolve the powder. Upon dissolution, 1.0 M KOH was added to raise the pH to 3.3–3.6. The solution was stored at 4 °C until use.

### 3.9. Stability Analysis

High-performance liquid chromatography (HPLC) with ultraviolet (UV) detection (Agilent 1100/1200 with diode array detector; Santa Clara, CA, USA) was used to assay BDQ samples for stability upon storage as either a dry powder or as a prepared solution. BDQ powders were stored at room temperature with desiccant, and BDQ solutions were stored at 4 °C. BDQ standards and samples (10 µL) were injected onto a Zorbax SB-C8 column (4.6 × 150 mm; 3.5-µm particles) with a flow rate of 0.8 mL/min and gradient from 95:5 water:acetonitrile (both solvents containing 0.015% perfluorobutanoic acid and 0.05% pentafluoropropionic acid) to 0:100 water:acetonitrile over 25 min. Elution was monitored at a wavelength of 265 nm. The limit of detection and limit of quantification of the method was 0.137 µg/mL and 0.458 µg/mL, respectively.

### 3.10. In Vivo Pharmacokinetic Study

All animal studies were approved by the Institutional Animal Care and Use Committee at Colorado State University. Dose-dependent efficacy with oral BDQ administration in M. tuberculosis-infected guinea pigs has been previously reported, with the dose at 15 mg/kg demonstrating the most potent reduction in bacterial burden [40]. This PK study was performed to evaluate the delivery of BDQ by intrapulmonary administration in two formulations, either dry powder or liquid. Guinea pigs were purchased with indwelling jugular venous catheters (Charles River, Canada) and maintained with twice daily flushing using 100 U/mL heparin sulfate in normal saline. Blood was collected using a 3-syringe technique, including sequential removal of 1 mL of blood from the catheter twice, followed by collection of 1 mL of blood for serum isolation and analysis. The initial 2 mL of blood was then returned to the animal, and the catheter was flushed with heparinized saline. This process was repeated for each blood collection time point. Blood was sampled at time 0 immediately prior to the administration of 15 mg/kg BDQ in powder or liquid formulation using a custom RTI International dosator or Penn Century microsprayer device, respectively. The serum was then collected at 0.5, 1, 2, 3, 6, 8, and 24 h after drug delivery for analysis via LC-MS/MS. Bioanalysis was performed by Cyprotex, LLC (Watertown, MA, USA).

### 3.11. Statistical Analysis

Significance testing for the in vivo PK study was performed using a 2-tailed Student’s *t*-test. Significance levels are denoted: * *p* < 0.05, ** *p* < 0.01, *** *p* < 0.005.

## 4. Conclusions

A respirable BDQ dry powder aerosol was successfully spray dried without the aid of an inert loop, increasing the accessibility of this manufacturing method for BDQ applications. The inclusion of the excipient leucine substantially enhanced the aerodynamic properties of the spray-dried powder. Using custom dosators, SD-BDQ-LEU was effectively administered as a dry powder to guinea pigs, the widely accepted animal model of tuberculosis, for the first time. The predominantly amorphous nature of the SD-BDQ-LEU product is not only useful for inhalation but aided in enhancing the solubility of BDQ for other delivery methods, such as liquid pulmonary instillation. Additional solubility enhancements were made using the excipient 2-hydroxypropyl-β-cyclodextrin, which allowed for the preparation of a 15 mg/mL BDQ aqueous solution despite BDQ’s inherent water insolubility. This aqueous solution was evaluated in guinea pigs, demonstrating increased serum concentrations compared to those achieved with the dry powder aerosol. An efficacy study utilizing spray-dried BDQ for inhaled powder and liquid delivery for the treatment of tuberculosis is currently underway.

## Data Availability

The data presented in this study are available on request from the corresponding authors.

## References

[1] World Health Organization (2022). Global Tuberculosis Report 2022.

[2] Chahine E.B., Karaoui L.R., Mansour H. (2014). Bedaquiline: A novel diarylquinoline for multidrug-resistant tuberculosis. Ann. Pharmacother..

[3] Li Y., Sun F., Zhang W. (2019). Bedaquiline and delamanid in the treatment of multidrug-resistant tuberculosis: Promising but challenging. Drug Dev. Res..

[4] Momin M.A.M., Rangnekar B., Larson I., Sinha S., Das S.C. (2019). Dry powder formulation combining bedaquiline with pyrazinamide for latent and drug-resistant tuberculosis. Adv. Powder Technol..

[5] Diacon A.H., Pym A., Grobusch M., Patienta R., Rustomjee R., Page-Shipp L., Pistorius C., Krause R., Bogoshi M., Churchyard G. (2009). The diarylquinoline TMC207 for multidrug-resistant tuberculosis. N. Engl. J. Med..

[6] Fox G.J., Menzies D. (2013). A review of the evidence for using bedaquiline (TMC207) to treat multi-drug resistant tuberculosis. Infect. Dis. Ther..

[7] Field S.K. (2015). Bedaquiline for the treatment of multidrug-resistant tuberculosis: Great promise or disappointment. Ther. Adv. Chronic Dis..

[8] Diacon A.H., Pym A., Grobusch M.P., de los Rios J.M., Gotuzzo E., Vasilyeva I., Leimane V., Andries K., Bakare N., De Marez T. (2014). Multidrug-resistant tuberculosis and culture conversion with bedaquiline. N. Engl. J. Med..

[9] Chesov D., Heyckendorf J., Alexandru S., Donica A., Chesov E., Reiman M., Crudu V., Botnaru V., Lange C. (2020). Impact of bedaquiline on treatment outcomes of multidrug-resistant tuberculosis in a high-burden country. Eur. Respir. J..

[10] Pontali E., Sotgiu G., D’Ambrosio L., Centis R., Battista Migliori G. (2016). Bedaquiline and multidrug-resistant tuberculosis: A systematic and critical analysis of the evidence. Eur. Respir. J..

[11] Zhao Y., Li F., Liu Y., Shi Y., Li Z., Cao G., Zhu W. (2018). Comparison of efficacy of inhaled and intravenous corticosteroid on pregnant women with COPD and the effects on the expression of PCT and hs-CRP. Exp. Ther. Med..

[12] Borghardt J.M., Kloft C., Sharma A. (2018). Inhaled therapy in respiratory diseases: The complex interplay of pulmonary kinetic processes. Can. Respir. J..

[13] Videira M.A., Llop J., Sousa C., Kreutzer B., Cossio U., Forbes B., Vieira I., Gil N., Silva-Lima B. (2020). Pulmonary administration: Strengthening the value of therapeutic proximity. Front. Med..

[14] Rawal T., Patel S., Butani S. (2018). Chitosan nanoparticles as a promising approach for pulmonary delivery of bedaquiline. Eur. J. Pharm. Sci..

[15] Maloney S.E., Mecham J.B., Hickey A.J. (2023). Performance Testing for Dry Powder Inhaler Products: Towards Clinical Relevance. KONA Powder Part. J..

[16] Momin M.A.M., Rangnekar B., Sinha S., Cheung C.-Y., Cook G.M., Das S.C. (2019). Inhalable dry powder of bedaquiline for pulmonary tuberculosis: In vitro physicochemical characterization, antimicrobial activity and safety studies. Pharmaceutics.

[17] Rangnekar B., Momin M.A.M., Eedara B.B., Sinha S., Das S.C. (2019). Bedaquiline containing triple combination powder for inhalation to treat drug-resistant tuberculosis. Int. J. Pharm..

[18] Okezue M.A., Byrn S.J., Clase K.L. (2022). Determining the solubilities for benzoate, nicotinate, hydrochloride, and malonate salts of bedaquiline. Int. J. Pharm..

[19] BUCHI Labortechnik AG (2020). B-290 Mini Spray Dryer Operation Manual.

[20] Alhajj N., O’Reilly N.J., Cathcart H. (2021). Leucine as an excipient in spray dried powder for inhalation. Drug Discov. Today.

[21] Seville P.C., Learoyd T.P., Li H.Y., Williamson I.J., Birchall J.C. (2007). Amino acid-modified spray-dried powders with enhanced aerosolization properties for pulmonary drug delivery. Powder Technol..

[22] Wolfenden R., Andersson L., Cullis P.M., Southgate C.C.B. (1981). Affinities of amino acid side chains for solvent water. Biochemistry.

[23] Glinski J., Chavepeyer G., Platten J.K. (2000). Surface properties of aqeuous solutions of L-leucine. Biophys. Chem..

[24] Chew N.Y., Chan H.K. (2001). Use of solid corrugated particles to enhance powder aerosol performance. Pharm. Res..

[25] Peng T., Lin S., Niu B., Wang X., Huang Y., Zhang X., Li G., Pan X., Wu C. (2016). Influence of physical properties of carrier on the performance of dry powder inhalers. Acta Pharm. Sin. B.

[26] Weiler C., Egen M., Trunk M., Langguth P. (2010). Force control and powder dispersibility of spray dried particles for inhalation. J. Pharm. Sci..

[27] Wu X., Li X., Mansour H.M. (2010). Surface analytical techniques in solid-state particle characterization for predicting performance in dry powder inhalers. KONA Powder Part. J..

[28] Chaurasiya B., Zhao Y.-Y. (2021). Dry powder for pulmonary delivery: A comprehensive review. Pharmaceutics.

[29] Stewart I.E., Lukka P.B., Liu J., Meibohm B., Gonzalez-Juarrero M., Braunstein M.S., Lee R.E., Hickey A.J. (2019). Development and characterization of a dry powder formulation for anti-tuberculosis drug spectinamide 1599. Pharm. Res..

[30] Maloney S.E., Alshiraihi I.M., Singh A., Stewart I.E., Mariner Gonzalez J., Gonzalez-Juarrero M., Meibohm B., Hickey A.J. (2023). Spray dried tigecycline dry powder aerosols for the treatment of nontuberculous mycobacterial pulmonary infections. Tuberculosis.

[31] Stewart I.E., Durham P.G., Sittenauer J.M., Barreda A.P., Stowell G.W., Moody C., Mecham J.B., Simpson C., Daily S., Maloney S.E. (2022). Optimization and scale up of spray dried CPZEN-45 aerosol powders for inhaled tuberculosis treatment. Pharm. Res..

[32] Maa Y.-F., Nguyen P.-A., Andya J.D., Dasovich N., Sweeney T.D., Shire S.J., Hsu C.C. (1998). Effect of spray drying and subsequent processing conditions on residual mositure content and physical/biochemical stability of protein inhalation powders. Pharm. Res..

[33] Okezue M., Bogdanowich-Knipp S., Smith D., Zeller M., Byrn S., Smith P., Purcell D.K., Clase K. (2021). Salts and Polymorph Screens for Bedaquiline. AAPS PharmSciTech.

[34] Shetty N., Cipolla D., Park H., Zhou Q.T. (2020). Physical stability of dry powder inhaler formulations. Expert Opin. Drug Deliv..

[35] Chen L., Okuda T., Lu X.-Y., Chan H.-K. (2016). Amorphous powders for inhalation drug delivery. Adv. Drug Deliv. Rev..

[36] Chang R.Y.K., Chen L., Chen D., Chan H.-K. (2020). Overcoming challenges for development of amorphous powders for inhalation. Expert Opin. Drug Deliv..

[37] Andries K., Verhasselt P., Guillemont J., Gohlmann H.W.H., Neefs J.-M., Winkler H., Van Gestel J., Timmerman P., Zhu M., Lee E. (2005). A diarylquinoline drug active on the ATP synthase of *Mycobacterium tuberculosis*. Science.

[38] Hirayama F., Uekama K. (1999). Cyclodextrin-based controlled drug release system. Adv. Drug Deliv. Rev..

[39] Vyas A., Saraf S., Saraf S. (2008). Cyclodextrin based novel drug delivery systems. J. Incl. Phenom. Macrocycl. Chem..

[40] Lenaerts A.J., Hoff D., Aly S., Ehlers S., Andries K., Cantarero L., Orme I.M., Basaraba R.J. (2007). Location of persisting mycobacteria in a guinea pig model of tuberculosis revealed by R207910. Antimicrob. Agents Chemother..

[41] Durham P.G., Hanif S.N., Contreras L.G., Young E.F., Braunstein M.S., Hickey A.J. (2017). Disposable dosators for pulmonary insufflation of therapeutic agents to small animals. JoVE.

